# National Working Group on the RE-AIM Planning and Evaluation Framework: Goals, Resources, and Future Directions

**DOI:** 10.3389/fpubh.2019.00390

**Published:** 2020-01-10

**Authors:** Samantha M. Harden, Thomas Edward Strayer, Matthew Lee Smith, Bridget Gaglio, Marcia G. Ory, Borsika Rabin, Paul A. Estabrooks, Russell E. Glasgow

**Affiliations:** ^1^Physical Activity Research and Community Implementation, Human Nutrition, Foods, and Exercise, Virginia Tech, Blacksburg, VA, United States; ^2^Translational Biology, Medicine, and Health, Virginia Tech, Blacksburg, VA, United States; ^3^Center for Population Health and Aging, Texas A&M University, College Station, TX, United States; ^4^Department of Environmental and Occupational Health, School of Public Health, Texas A&M University, College Station, TX, United States; ^5^Department of Health Promotion and Behavior, College of Public Health, University of Georgia, Athens, GA, United States; ^6^Clinical Effectiveness and Decision Science Program, Patient-Centered Outcomes Research Institute, Washington, DC, United States; ^7^Department of Family Medicine and Public Health, School of Medicine, University of California, San Diego, San Diego, CA, United States; ^8^Adult and Child Consortium for Outcomes Research and Delivery Science Dissemination and Implementation Science Program and The Department of Family Medicine, School of Medicine, University of Colorado- Anschutz Medical Campus, Aurora, CO, United States; ^9^Eastern Colorado QUERI and GRECC Programs, University of Colorado- Anschutz Medical Campus, Aurora, CO, United States; ^10^College of Public Health, University of Nebraska Medical Center, Omaha, NE, United States

**Keywords:** dissemination, implementation, resources, application, synopsis

## Abstract

The National Working Group on RE-AIM Planning and Evaluation Framework (herein Workgroup) was established in 2004 to support the application of the framework and advance dissemination and implementation science (D&I). Workgroup members developed and disseminated products and resources (and continue to do so) to advocate for consistent application of RE-AIM and allow for cross study comparisons. The purpose of this paper is to summarize key Workgroup activities, products, and services (e.g., webinars, consultations, planning tools) and enhance bidirectional communication between the Workgroup and RE-AIM users. The ultimate goal of this work is to serve as a forum for dissemination to improve the balance between RE-AIM user demand (needs) and the currently limited RE-AIM Workgroup supply (consultation and resources) to demonstrate and expand the utility of RE-AIM as a D&I planning and evaluation framework. A summary of resources is provided as well as specific examples of how the Workgroup has been responsive to user needs.

## Introduction and History

Dissemination and implementation (D&I) research is designed to facilitate integration of research into practice and policy and study the process by which this occurs (1). D&I science includes a number of models, theories, frameworks (herein models) to guide D&I science activities that have become much more prominent in recent years (2, 3). However, researchers often struggle operationalizing these models in their work (3). Resources and guidance are needed to support both researchers and practitioners to apply D&I models to their work in a meaningful way. In response to this need, a number of training programs were developed to support immersion in D&I models, methods, and measures (4). These trainings typically combine in person and virtual meetings, mentorship, reading lists, and webinars. Evaluations of these trainings have been positive, but they have limited resources (i.e., mentors, funding, time) and therefore, limited reach among people who wish to apply D&I models in practice. To respond to these demands, several research groups provide a suite of materials to introduce and guide the use of specific D&I models (5–8). The suite of options typically showcases websites, webinars, static materials, and opportunities for consultation (7).

This suite of D&I model resources should have capabilities to reach and be useful to a large number of researchers and, of equal or greater importance, be understandable and useable for practitioners and healthcare teams. These resources should also provide user support to ensure adoption, consistent application of ideas, and, when necessary, appropriate adaptation of model implementation. This paper is centered on one of the most frequently used (9–11) D&I models, RE-AIM (reach, effectiveness, adoption, implementation, and maintenance) (12) and the various activities conducted since 2004 to provide general and tailored guidance and support for the operationalization of RE-AIM.

RE-AIM was developed in the late 1990s with the intent to support the translation of research to practice and policy, to improve the impact of health promotion and prevention efforts (4, 9, 12–14). The RE-AIM website, initially funded by the Robert Wood Johnson Foundation, was developed in 2004 to enhance understanding and transparency of the framework, provide application resources and address commonly asked questions (8). The website has consistently included RE-AIM-relevant papers, presentations, definitions, and checklists. Over the years, interactive materials have been developed including self-quizzes with feedback and suggestions, webinar viewings, and RE-AIM calculators. With limited funding, the website has been continuously supported by RE-AIM investigators, called the National Working Group on RE-AIM Planning and Evaluation Framework (herein the Workgroup). Members of the Workgroup consist of senior investigators who have applied RE-AIM since its inception and early career researchers who studied and applied RE-AIM in graduate school. This Workgroup is similar to other research or professional networks; membership is not exclusive but rather driven by shared tasks and mission (15–17).

The purpose of this paper is to summarize key activities, products, and services (e.g., webinars and planning tools) that the Workgroup offers. By doing so, Workgroup members invite RE-AIM users to provide feedback and recommend additional resources that could contribute to advancing D&I science. Some key efforts of the Workgroup are summarized including: (1) website resources, evolution, and future directions; (2) papers, presentations, and webinars; and (3) consultation experiences and offerings (e.g., grant consultations, trainings, and practice facilitation). It is our intent to connect those applying RE-AIM in clinical, corporate, or community settings with necessary resources, and also inspire others to apply the model, share new applications, and communicate additional needs. The ultimate goal is to serve as a forum for dissemination (18) to improve the balance between RE-AIM user demand (needs) and the RE-AIM Workgroup supply (offerings) to promote usefulness of RE-AIM as a D&I planning and evaluation framework.

## Goals of the Workgroup

The mission of the RE-AIM Workgroup is to implement a robust and evolving framework to advance science, enhance practice, and influence policy through collaboration and training. In addition to collaborative research to advance RE-AIM, members provide guidance for its use (particularly as related to its evolution in response to new settings, purposes, opportunities, data, and challenges) (9), In a recent summary of the RE-AIM framework over its 20 year history (9), published by the Workgroup, a number of suggestions for future use of RE-AIM were put forth. These included (1) enhanced reporting and evaluation of implementation context and strategies; (2) application of mixed-methods research designs; (3) more rapid and iterative use of RE-AIM; and (4) combining RE-AIM with other relevant D&I and pragmatic frameworks and approaches such as the Pragmatic Explanatory Continuum Indicator Summary (PRECIS-2) model (19, 20). These suggestions were, in part, in response to the needs of RE-AIM users shared with members of the Workgroup—via email, in person conversations, or website requests. By way of example, one Workgroup member recently consulted on a project reviewing physical activity self-management for patients with spinal cord injury that applied both PRECIS-2 and RE-AIM (21). This led to development of a new data extraction tool, discussions among co-authors on the nuances of the RE-AIM framework, and swift responses to peer review journal manuscript reviewer comments. To further reporting and evaluation, two members of the Workgroup completed a study to qualitatively identify and assess the planning, implementation, evaluation, and dissemination using RE-AM and the Practical Robust Implementation and Sustainability Model (PRISM) extension of the framework across four health services intervention projects in the Veterans health administration setting. PRISM is a contextual expansion of the RE-AIM framework which includes theoretical constructs hypothesized to be predictive of each of the RE-AIM dimensions. The constructs include intervention characteristics, recipient characteristics, implementation and sustainability infrastructure, and external environment domains (22, 23). Results of the Veterans health administration projects pointed to the need to engage key stakeholders, assess how an intervention “fits” the targeted system, and to adjust/adapt over time and for different settings as keys for the success of dissemination and implementation efforts (23). An ongoing effort of a sub-group of Workgroup members is using a protocol to apply RE-AIM iteratively to inform adaptations during the implementation of health services interventions across four health services research projects. This effort starts with periodic evaluative reflections by the implementation team for the project including ratings on the importance of and progress on the various RE-AIM dimensions. This exercise is followed by the selection of and goal setting around one to two RE-AIM dimensions that are most important at the given time of the project and could benefit most from improvement. Examples include the selection of and goal setting on RE-AIM dimensions of (a) “reach,” with activities identified as better specification of target audience or eligible population at each implementation site, outreach to sites to assess barriers for increasing recruitment of eligible participants; and (b) “effectiveness,” to align original implementation and clinical outcomes to better match changed organizational priorities.

## Resources and Activities Supported by the Workgroup

### Overview of the RE-AIM Website

**Figure 1:**
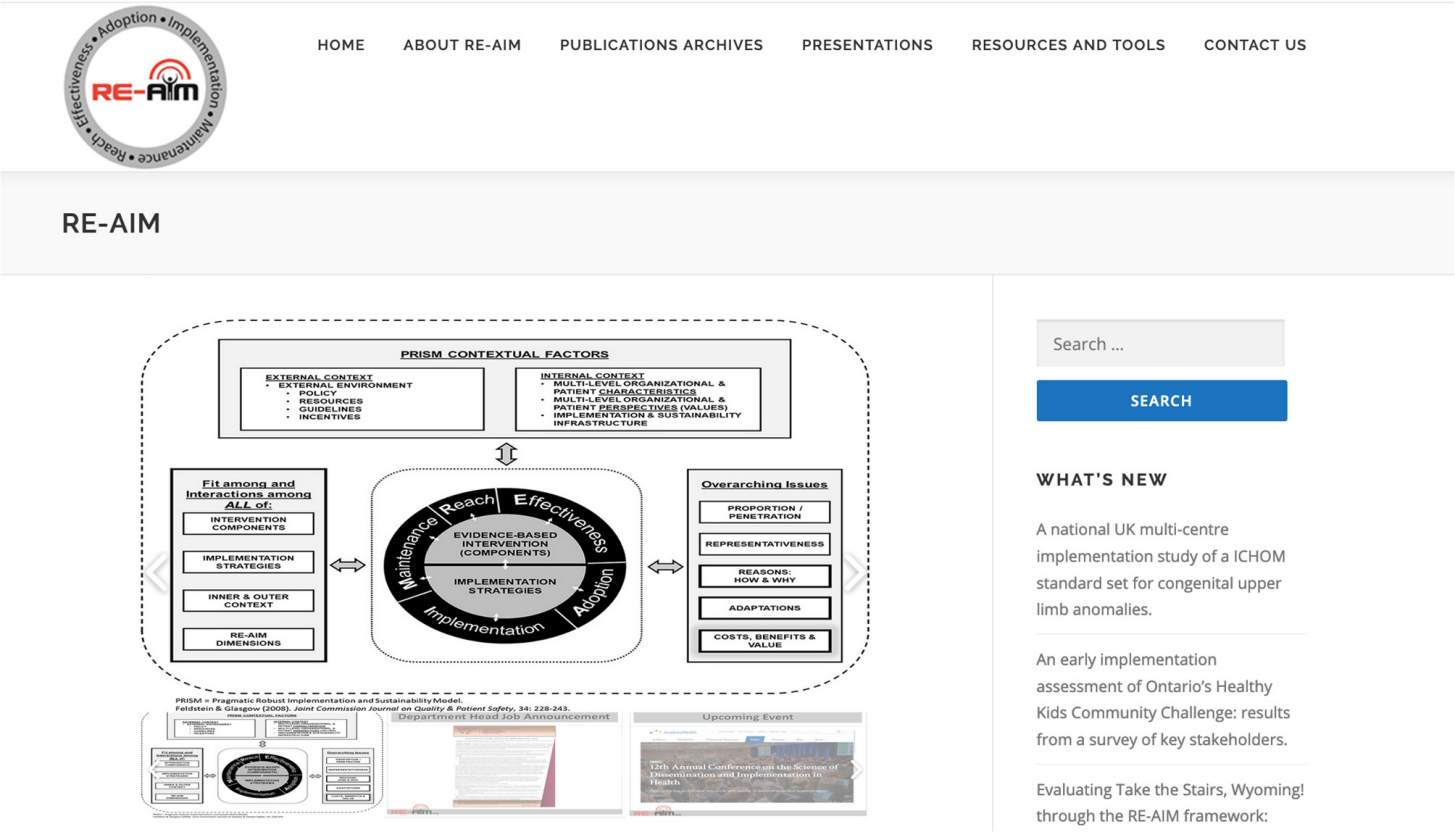
http://www.RE-AIM.org website layout.

The purpose of the Workgroup website (www.RE-AIM.org) (8) is to support and connect those in need of explanation or resources to apply the RE-AIM framework. Specifically, the Workgroup website introduces the framework and supports implementation researchers and practitioners with guidance on application and reporting of framework dimensions. The site contains a collection of tools as well as contextual and measurement considerations when using RE-AIM in implementation research (see Figure 1 for illustration of website homepage). For example, it has a full description of each RE-AIM dimension, the methods to conduct research and planning for each, and publications about how these methodologies are applied. Finally, the website serves as an intermediary to connect users to other implementation resources such as the National Cancer Institute's Research-Tested Intervention Programs (RTIPs) and Implementation Science websites (24).

### Who Is Using the Website?

Google analytics software was added to the RE-AIM site in January of 2015. Data are tracked to monitor the number of sessions started, unique users who access the website, the number of page views, average time for each session, and which pages have the most visits and downloads. From January 2015 to March of 2017, these data were reported quarterly, and since April of 2017, they have been reported monthly. In 2015, there were 3,387 unique users and 3,531 sessions initiated. The website usage has increased over time and in 2018, there were 32,793 unique users (36% increase from 2017) and 48,236 (31% increase from 2017) sessions. Since early 2017, the majority of the sessions, come from the United States (69%), followed by Australia (19%), United Kingdom (9%), Canada (9%), Netherlands (4%), and Germany (2%).

### Page Visits

Most users start with the homepage (Figure 1), the “What is the RE-AIM framework?” page or the “Frequently Asked Questions” page. The next most frequently visited resources are the “Measures and Checklists” and “Applying the RE-AIM Framework” pages. Unfortunately, there are no current means to capture patterns/pathways of navigation of RE-AIM site users, though understanding this information could aid in improving the user experience.

### Resources, Tools, and Guides

A variety of both static (stand-still, downloads) and interactive (listserv, webinars, planning tools) resources are provided on the site. The website includes links to other relevant D&I resources such as cfir.org (Consolidated Framework for Implementation Research website), reporting guidelines from CONSORT (25, 26), Standards for Reporting Implementation Studies (StaRI) Statement (27), and ways for site users to find evidence-based interventions [e.g., through Research Tested Intervention Programs (RTIPs)]. Another primary resource is the RE-AIM Planning and Evaluation Tool, which was iteratively developed to assist users from various sectors (e.g., research, public health, healthcare) to consider the RE-AIM dimensions and enhance implementation over time. Launched in June 2017, the updated planning tool is a portable document format (PDF) instrument organized to provide prompts for consideration related to common challenges to successful application of RE-AIM. The tool prompts users to consider to whom the initiative will appeal (Whom do you plan to reach and How do you define the intended beneficiaries?). For effectiveness, the planning tool prompts users to consider: “What might be the unintended consequences or outcomes? What has gone wrong in other similar initiatives?” An adapted version of these considerations is presented in a manuscript outlining the iterative use of RE-AIM before, during, and after intervention implementation (28). A detailed example of applying the planning tool across clinical, corporate, and community settings can be found in Harden et al. (28).

### RE-AIM Publication Tracking and Repository

**Figure 2:**
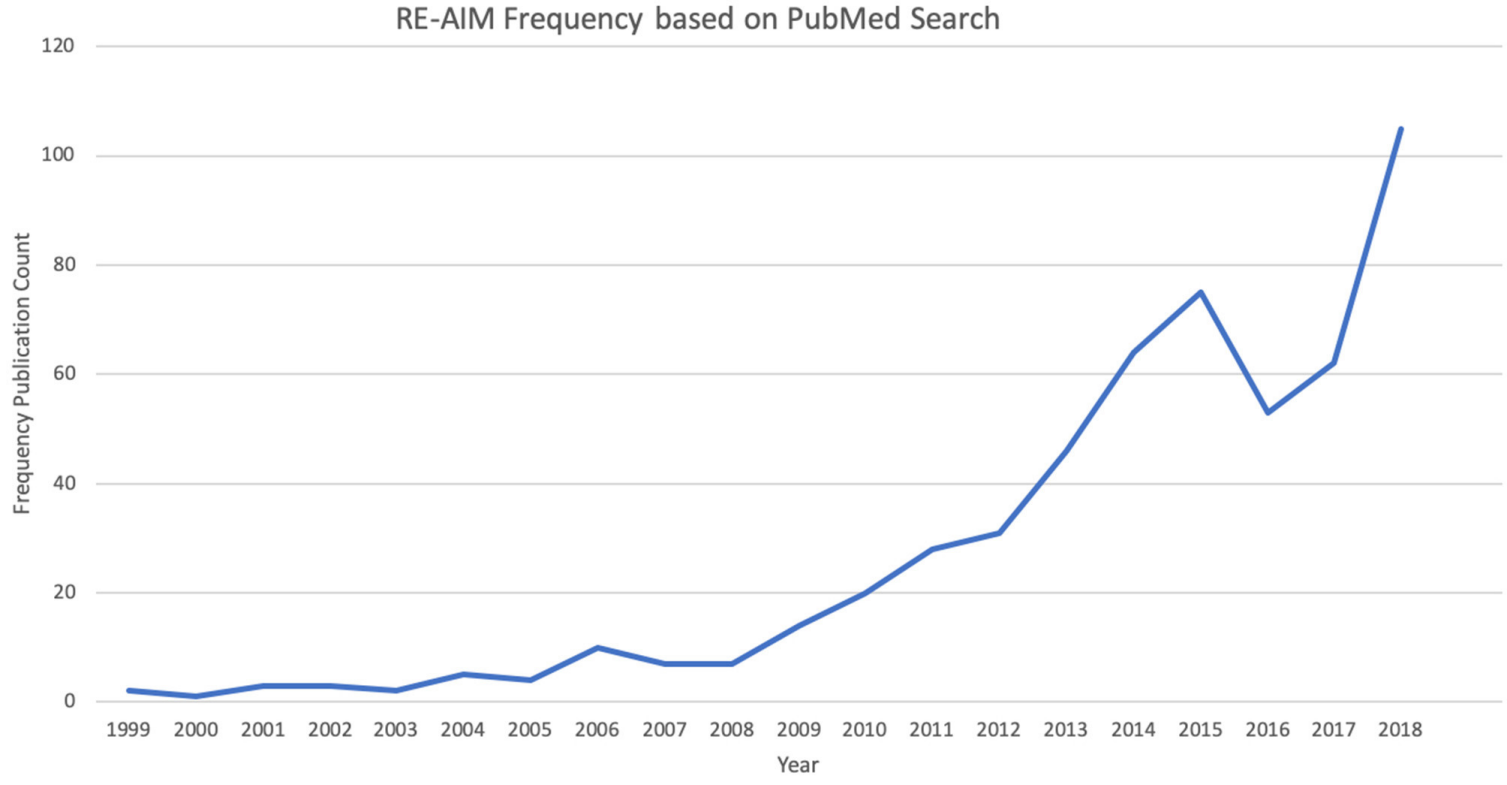
Reach, Effectiveness, Adoption, Implementation, and Maintenance (RE-AIM) Framework Peer-Review Publications—frequency by year (1999–2018).

A graduate research assistant conducts a monthly PubMed search for “RE-AIM” so users can better understand the breadth, scope and content of RE-AIM publications. See Figure 2 for the number of publications on RE-AIM, by year. In total, the RE-AIM framework has appeared in over 500 publications since its initial publication in 1999, with vastly enhanced publication rate beginning in 2009. The website includes a search feature for users to identify papers through keyword search, such as topic or target audience (e.g., physical activity, youth).

## Webinar Series

The RE-AIM Workgroup and website also host and post recorded webinars featuring speakers that discuss the use of RE-AIM in research and in practice, and address questions from the audience. Each webinar is ~1 h in duration, and usually follows the format of having a moderator introduce the topic, prepared remarks from the main speaker, reflections from other Workgroup members, and time for open audience questions and discussion. From 2015 to present, there have been 27 webinars with an average of 26 live audience attendees. One of the most popular webinars to date, was delivered in January of 2018. It was titled “Reflections from the field: RE-AIMers reflect on annual D&I meeting” (10th Annual Conference on the Science of Dissemination and Implementation in Health) with panelists sharing their top lessons learned, experiences, and tips for the annual meeting.

## Consultation and Presentations

Members of the Workgroup have provided a number of tutorials and workshops on the application of RE-AIM for target outlets such as the Society of Behavioral Medicine (sbm.org) and American Public Health Association (apha.org), national non-profit and federal grantee meetings in aging and public health. Members of the Workgroup also provide educational workshops, guest lectures within the U.S. and internationally (in person or via the online platform Zoom) to a variety of audiences (e.g., undergraduate kinesiology students, graduate implementation science courses, grantee meetings, and professional forums with service organizations) and on-line technical assistance (please see website under the publications and presentations sections for examples of these efforts). While not unique to the RE-AIM framework specifically, many Workgroup members are included as co-investigators or consultants on grant proposals and funded projects to bolster the application of RE-AIM. An early example was the “adoption” of the RE-AIM framework by the Centers for Disease Control and Prevention Healthy Aging Research Network as a way of understanding the impact of promoting physical activity programs for older adults (29). Workgroup members have advised national funding agencies on the use of the RE-AIM framework leading to its recommended or required inclusion in the grant applications as well as technical assistance to grantees in its practical application (30).

## Publications

Complementing the more than 500 publications referencing the RE-AIM framework, core Workgroup members have recently published two key collaborative summary papers. One paper, describes the past, present, and future application of the RE-AIM framework (9). The other describes the iterative application of RE-AIM in clinical, corporate, and community settings (28). Workgroup members have also collaborated on the application of RE-AIM in a multitude of research projects and publications and provided crosscutting resources including: (1) several literature reviews of use of RE-AIM in different content areas and settings (10, 13, 31, 32); (2) how to operationalize RE-AIM in pragmatic and more practitioner-friendly language of who, what, where, when, why, and how (13); (3) use of RE-AIM to evaluate statewide walking programs in Extension (33); and (4) editing a RE-AIM-based Research Topic in the journal Frontiers in Public Health.

This wide array of publications demonstrates the utility of the framework to address scientific questions across a variety of dissemination and implementation outcomes. Specifically, the framework has been used to guide the development and assessment of interventions that expand beyond simply improving effectiveness—to include an explicit focus on improving individual and organizational-level dissemination (34, 35) of evidence-based approaches (i.e., reach and adoption, respectively) and improving implementation quality, costs, and likelihood of organizational sustainability (36, 37). It is also possible to categorize publications by different levels on a translational research spectrum from efficacy (38), to effectiveness (39), to dissemination (35), to sustainability (40) across a variety of intervention types—program, policy, systems, and environmental changes (41–43). Indeed, the accumulation of literature demonstrating the utility of the framework matches the promise to improve planning, evaluation, and scientific advancement in health promotion of early RE-AIM articles (44–46).

## Future Directions

Members of the National Working Group on RE-AIM Planning and Evaluation Framework are committed to advancing D&I science through the rigorous application of the framework and related approaches across a wide variety of research areas. This involves continuously evaluating the framework's utility for planning and assessing different interventions and implementation strategies, and in diverse populations and settings. We are also continuing to push the boundaries of the framework and test its scope of applicability, and are open to making modifications to address evolving issues (9). Such D&I work is notably complex, but critical to understand a program or policy's ability to be easily adopted and adapted, reach those most in need, ensure that it is delivered with fidelity, and that it can produce replicable and long-lasting individual and systems-level improvements.

Shoup and colleagues' network analysis of RE-AIM framework use commented that researchers publishing on RE-AIM were part of an “invisible college” (47). Since 2004, members of this well-connected RE-AIM college have worked to ensure that RE-AIM use is not restricted to a small set of individuals, but instead focused on sharing resources, experiences, and novel applications of the framework. The RE-AIM website and its offerings (webinars, consultations, tools, and resources) are one strategy to disseminate information and seek two-way communication with RE-AIM users. Researchers and practitioners are encouraged to contact the Workgroup regarding resources and needs through the Website (http://www.re-aim.org/contact/).

The Workgroup has focused on making RE-AIM accessible and adaptable across contexts including clinical, community, and other pragmatic settings (28). To accomplish this flexibility, the Workgroup is open to feedback through a variety of formats including contact tools on the RE-AIM website, active presence at conferences, webinars, and presentations.

To support the new research and practice directions summarized above (e.g., enhanced evaluation of contextual factors impacting RE-AIM dimensions; increased application of mixed-methods approaches; more rapid and iterative use of RE-AIM; and combining RE-AIM with other relevant D&I and pragmatic frameworks) we anticipate providing additional resources, application guides, new website features, and more concrete examples of new uses of RE-AIM.

We welcome reader input on these directions and resulting new resource needs. Designing features based on user feedback should enhance the usefulness of the framework and its website (48, 49).

## Conclusions

This article summarizes progress by the RE-AIM Workgroup and the open-access resources that the workgroup has developed. The use of RE-AIM has moved beyond its original intent—to improve how programs are evaluated—to being a cornerstone for how programs and research are planned and evaluated, including the implementation phase. The continued popularity of RE-AIM can be attributed to the applicability of the core tenets of the RE-AIM framework across population and settings, its relative ease of use, and understandability to stakeholders (30). We do not see RE-AIM as static, but anticipate that the framework will continue to evolve based on advances in D&I science, to meet user needs and address new applications. For the last 10 years, Workgroup members have used limited resources to ensure that the RE-AIM framework is disseminated to researchers and practitioners. That said, there are ongoing opportunities to enhance the resources, tools and guides provided; therefore, through this paper and our mission, Workgroup members encourage users to take a proactive part in RE-AIM's continual evolution.

## Author Contributions

All authors contributed to the conceptualization of the manuscript and its content. All authors contributed to the full manuscript as well as reviewed and approved the final version of the manuscript.

### Conflict of Interest

Co-authors are members of the National Working Group on RE-AIM Planning and Evaluation Framework (www.re-aim.org). The authors declare that the research was conducted in the absence of any commercial or financial relationships that could be construed as a potential conflict of interest.
